# Supplementary material for: “It Would Ruin My Life”: Pacific Islander Male Adolescents’ Perceptions of Mental Health Help-Seeking—An Interpretative Phenomenological Analysis Focus Group Study
Source: Int J Environ Res Public Health. 2025 Jan 4;22(1):62. doi: 10.3390/ijerph22010062 (PMC11764690; doi:10.3390/ijerph22010062)
Supplement: Supplementary file 1 [file ijerph-22-00062-s001.zip › ijerph-3386107-supplementary.pdf]

Interview Protocol Questions

Question 1:

Format: Open-ended

How would you respond if a family member or friend in the Polynesian/Pacific Islander community told you they were struggling with a mental health concern?

Question 2:

Format: Open-ended

If you were to recommend that your friend/family member seeks help, where would you recommend that they go?

Question 3:

Format: Card-selection

Materials Needed: One pre-printed deck of cards for each participant

3a) Here are some other resources available to address mental health concerns. Pick 5 cards that you feel would be the most helpful. You may also use the blank cards to write down other resources that you may not see on the table.

- Professional therapist or counselor
- Close family member
- Relative
- Colleague/co-worker
- Roommate/housemate
- Supervisor/boss
- Church member
- Church official (bishop, pastor, clergyman, etc.)
- Doctor or physician
- Friend
- Neighbor
- Teacher or professor
- School counselor
- Tribal elder
- Cultural healer
- Acquaintance
- Stranger
- Other medical professional: List here

3b) Please sort these mental health resources into most helpful, somewhat helpful, and not helpful

Format: Card Sort and Follow up Questions as necessary

Materials:

1. One set of sorting guides labeled with:
    a. Definitely helpful
    b. Most likely helpful,
    c. Possibly Helpful;
    d. Not at all helpful.

2. Another set of cards with the below words printed on them (also remember to bring blank cards to reproduce what participants may add to the resource list):

- Professional therapist or counselor
- Close family member
- Relative
- Colleague/co-worker
- Roommate/housemate
- Supervisor/boss
- Church member
- Church official (bishop, pastor, clergyman, etc.)
- Doctor or physician
- Friend
- Neighbor
- Teacher or professor
- School counselor
- Tribal elder
- Cultural healer
- Acquaintance
- Stranger
- Other medical professional: List here

Talk with participants about how the resources were sorted. Consider probing words such as: ***I noticed ….Can you explain more? Can you give an example? Say more about that? Tell me more. Is there anything else. I'm not sure I understand – say more. Can you be more specific? When you say ____ what do you mean? Help me understand. Can you clarify? Can you please describe what you mean? Could you give me an everyday example?***

Break

Protocol: We are about to take a 20 minute break. Feel free to relax, use the restroom or get a snack.

Upon returning from the break: We'd like you to take a few minutes write down any thoughts or questions you may from our first session that we didn't get a chance to talk about yet.

Question 4: For the cards that you selected, what are some of the pros and cons to using each of these resources?

Format: Card-selection

Materials Needed: One pre-printed deck of cards for each participant

Question 5: If someone who identifies as being Polynesian American/South Pacific Islander sought out a mental health service or support, how would they be viewed in their community?

Format: Open Ended

Question 6: What would keep someone from reaching out to a professional?

Format: Card sort and Follow Up questions

Materials Needed:

1. One set of sorting guides preprinted:
    a. Would definitely keep someone from reaching out to a professional.
    b. Would most likely keep someone from reaching out to a professional;
    c. Would possibly keep someone from reaching out to a profession;
    d. Would not keep someone from reaching out to a professional.

2. One pre-printed deck of cards for each participant pre-printed with the following:

- Friends should be support – I only go to my friends
- Family is my support – I only go to my family
- Cultural Leader would not approve
- Family would not approve
- Wouldn't know where to go
- Cost too much
- I would be uncomfortable talking about my problems to someone else

- Shame/guilt
- Logistics
- Safety
- I would feel too embarrassed
- Stigma

Follow up questions: **I noticed….** ***Can you explain more? Can you give an example? Say more about that? Tell me more. Is there anything else? I'm not sure I understand – say more. Can you be more specific? When you say ____ what do you mean? Help me understand. Can you clarify? Can you please describe what you mean? Could you give me an everyday example?***
